# A New Remedy for Hiccough

**Published:** 1886-02

**Authors:** 


### A New Remedy for Hiccough.

Dr. O. T. Shultz, of Mt. Vernon, Indiana (*American Practitioner*), relates a case of hiccough of nine days' duration, which yielded on the morning of tenth day to one drop of a one per cent. solution of nitro-glycerine, and repeated in one hour. The remedy was continued at intervals of two hours—gradually extended. On the twelfth day permanent relief was obtained, and treatment discontinued.

